# Circulating Immune and Endocrine Markers in Currently Drinking and Abstinent Individuals With Alcohol Use Disorder and Controls

**DOI:** 10.1111/adb.70039

**Published:** 2025-05-02

**Authors:** Ryan E. Tyler, Carlotta Vizioli, Jennifer J. Barb, Mehdi Farokhnia, Lorenzo Leggio

**Affiliations:** ^1^ Clinical Psychoneuroendocrinology and Neuropsychopharmacology (CPN) Section, Translational Addiction Medicine Branch National Institute on Drug Abuse Intramural Research Program and National Institute on Alcohol Abuse and Alcoholism Division of Intramural Clinical and Biological Research, National Institutes of Health Baltimore Maryland USA; ^2^ National Institute of General Medical Sciences Bethesda Maryland USA; ^3^ Interoceptive Disorders Unit, Office of the Clinical Director National Institute of Neurological Disorders and Stroke, NIH Bethesda Maryland USA; ^4^ Translational Biobehavioral and Health Disparities Branch, Clinical Center, NIH Bethesda Maryland USA

**Keywords:** abstinence, biomarkers, BDNF, GLP‐1, IL‐8, IL‐18

## Abstract

Alcohol use disorder (AUD) is associated with changes in endocrine and immune system function. This study is a secondary analysis aimed at investigating changes in circulating immune and endocrine biomarkers in blood samples from three groups: (1) healthy controls (HC, *N* = 12), (2) AUD—currently drinking, nontreatment seeking (CD, *N* = 9), and (3) AUD—abstinent, treatment‐seeking (AB, *N* = 10; abstinent for at least 6 weeks). We hypothesized that both immune and endocrine biomarker concentrations would be different in AUD groups compared to healthy controls. Immune biomarkers included IL‐8, IL‐18, CCL2, TNF‐α, IL‐1RA, IL‐6, and IL‐10. Endocrine biomarkers included brain‐derived neurotrophic factor (BDNF), glucagon‐like peptide 1 (GLP‐1), ghrelin, gastric inhibitory peptide (GIP), growth hormone, leptin, and insulin. Biomarker concentrations were compared between the three groups while controlling for age and sex, and associations between biomarker concentrations and behavioral measures were explored. IL‐8 concentrations were elevated in AB compared to CD and HC (*F*(2,29) = 6.33, *p* = 0.006, ƞ_p_^2^ = 0.318). BDNF concentrations were lower in AB compared to HC (*F*(2,30) = 4.34, *p* = 0.02, ƞ_p_^2^ = 0.266). GLP‐1 concentrations were higher in AB compared to HC (*F*(2,25) = 4.22, *p* = 0.03, ƞ_p_^2^ = 0.287). Exploratory analyses in combined groups showed that measures of past drinking, AUD severity, and anxiety/depression positively correlated with IL‐18 and TNF‐α and negatively correlated with BDNF. These results demonstrate that circulating concentrations of both immune and endocrine proteins are altered in abstinent individuals with a history of severe AUD (AB group) compared to healthy controls. In contrast, no group differences were observed for any biomarker between the nontreatment seeking, currently drinking people with AUD and the HC group. Our findings highlight the importance of accounting for AUD severity, comorbidities, and treatment‐seeking status, especially when studying alcohol‐related biomarkers.

## Graphical abstract

Circulating concentrations of BDNF, GLP‐1 and IL‐8 were altered only in abstinent individuals with AUD, likely reflecting greater disease burden and comorbidities compared to current drinkers.

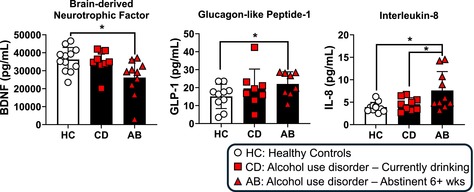

## 1 Introduction

Alcohol use disorder (AUD) is a chronic disease characterized, among other clinical features, by impaired control of alcohol use despite known negative consequences; AUD is associated with high morbidity and mortality [1]. There are currently only three FDA‐approved medications for AUD (naltrexone, acamprosate, and disulfiram), and most people with AUD do not receive any treatment [2]. Furthermore, there is a high degree of heterogeneity among people with AUD. These factors underscore the importance of developing novel treatment strategies. In addition to the nervous system, the immune and endocrine systems hold promise as potential pharmacotherapeutic targets for AUD [1, 3]. As such, there is a critical need to better understand the immune and endocrine system adaptations in the context of alcohol use and AUD.

The immune system is a distributed network of cells, tissues, and organs that primarily function to protect the body from pathogens. Alcohol use perturbs the immune system, and repeated and heavy alcohol use produces long‐term adaptations in the immune system [4, 5, 6, 7, 8, 9, 10]. One study found that oral alcohol administration resulted in decreased concentrations of circulating tumor necrosis factor α (TNF‐α) and increased concentrations of interleukin 6 (IL‐6) 3 h post consumption in people with AUD [9]. Some studies have found that alcohol use suppresses the immune system, resulting in increased susceptibility to infectious diseases [11]. Alcohol also damages the lining of the gut, allowing bacteria to enter the blood stream and activate an immune response [12]. Acute alcohol use resulted in decreased TNF‐α and increased IL‐6 blood concentrations when measured 3 h post alcohol consumption [9]. In addition to acute perturbations of the immune system, AUD is associated with lasting changes in the immune system. Blood markers of intestinal permeability and inflammation such as lipopolysaccharides (LPS), TNF‐α, IL‐6, IL‐10, and high‐sensitivity C‐reactive protein (hsCRP) were increased in patients with AUD but without cirrhosis, compared to healthy controls. Interestingly, much of the proinflammatory biomarkers were significantly downregulated after 3 weeks of alcohol abstinence [13]. Another study showed increased mRNA expression of TLR4, TLR2, CD14, MyD88, IL‐1β, IL‐8, IL‐18, and peptidoglycan protein concentrations in peripheral blood mononuclear cells (PBMCs) from blood samples of people with AUD compared to healthy controls [14]. More studies have found higher concentrations of TNF‐α and CRP in people with AUD compared to controls [15], as well as other studies demonstrating associations between early life stress, AUD, and inflammatory markers [16, 17]. Anti‐inflammatory markers, including IL‐10 and interleukin‐1 receptor antagonist (IL‐1RA), are also affected by alcohol. An *IL10* gene polymorphism was associated with AUD [18], and studies in animals indicate that IL‐10 may remediate the effects of alcohol exposure on amygdala GABA transmission and anxiety‐like behavior [19]. Another animal study found that treatment with IL‐1RA blunts alcohol‐induced liver steatosis, inflammation, and injury [20]. Furthermore, alcohol use is a leading cause of multiple organ failure, illustrating the damaging effects of alcohol on nearly all organs in the body [3, 21]. As such, for a comprehensive evaluation of immune system function, we tested a range of immune biomarkers: IL‐6, IL‐8, IL‐10, IL‐18, IL‐1RA, chemokine ligand 2 (CCL2, a.k.a. monocyte chemoattractant protein 1, MCP‐1), and TNF‐α.

The endocrine pathways include distributed networks of cells highly integrated with the body's other systems, including the immune and nervous systems. Endocrine glands release hormones into the blood stream that act on receptors throughout the body to regulate physiological functions and maintain homeostasis [22]. Many endocrine pathways are influenced by alcohol use and AUD, and some endocrine targets, especially metabolic and feeding‐related hormones, represent new pharmacotherapeutic targets for AUD given the overlapping mechanisms between feeding/satiety and regulation/craving of alcohol [3, 23, 24, 25, 26, 27, 28, 29, 30, 31]. One such endocrine biomarker is the brain‐derived neurotrophic factor (BDNF), which is essential for synaptic plasticity and is downregulated in people with AUD and negatively associated with drinking and AUD severity [32, 33, 34, 35, 36, 37]. Enhancing BDNF in animal models has been demonstrated to regulate glucose homeostasis in diabetic mice (db/db) [38, 39, 40, 41, 42] and regulates hedonic feeding through changes in mesolimbic dopaminergic pathways [43]. Furthermore, BDNF regulates insulin release by acting on pancreatic beta cells [44]. Insulin, the hormone that promotes glucose uptake into cells for cellular energy production, is also associated with AUD and alcohol craving [45, 46]. Additionally, leptin, an adipose‐derived hormone involved in satiety regulation, has shown altered gene polymorphisms and protein concentrations in people with AUD [47, 48]. Acute alcohol consumption has been demonstrated to increase plasma growth hormone (GH) concentrations [49], and people with AUD were less sensitive to pharmacologically induced GH secretion [50].

In addition to these hormones, incretin gut hormones like the gastric inhibitory peptide (GIP), glucagon‐like peptide 1 (GLP‐1), and ghrelin are also targets of interest in AUD [23, 24, 25, 26, 27, 51, 52].

As such, the present study evaluated different endocrine biomarkers with a focus on feeding‐related proteins for a comprehensive evaluation of endocrine dysfunction in AUD. The endocrine proteins tested in the present work include BDNF, insulin, GH, GIP, leptin, GLP‐1, and ghrelin.

Evidence suggests a close relationship between the immune, nervous, and endocrine systems [53, 54, 55, 56], and the dysregulation of these systems by alcohol is likely influenced by each other. For example, BDNF showed a strong negative correlation with multiple proinflammatory markers including IFN‐γ, IL‐1β, IL‐4, IL‐8 in people with early‐stage breast cancer [57], and BDNF has been shown to have direct anti‐inflammatory effects [58]. In addition to BDNF, gut hormones like GLP‐1 [59], ghrelin [60], and GIP [61, 62] have broad anti‐inflammatory effects. Ghrelin is the endogenous ligand of the growth hormone secretagogue receptor (GHSR), which results in GH secretion. Unsurprisingly, GH also has anti‐inflammatory effects [63]; however, GH can also promote inflammation depending on the context [64]. In addition, insulin and leptin are closely associated with immune function [65, 66, 67]. Such close relationships between the endocrine and immune systems led us to examine both in the present study to provide a comprehensive picture in the context of AUD.

AUD is a dynamic process with changes that may vary depending on current drinking or abstinence status. Further, acute pharmacological effects of alcohol in people currently drinking can heavily influence biomarker concentrations. As such, it is important to characterize biobehavioral correlates of AUD in people currently drinking and in abstinent individuals. The present study used a three‐group design: healthy control (HC) group, a group with AUD that had not received nor were currently seeking treatment and were currently drinking (CD), and a group with AUD that had recently completed inpatient treatment and were abstinent (AB) from alcohol for at least 6 weeks. We hypothesized that both immune and endocrine biomarker concentrations would be different in AUD groups compared to healthy controls, and that these changes would be associated with alcohol‐related behavioral measures. As such, in this secondary analysis, we compared circulating concentrations of immune and endocrine biomarkers from blood samples between the three groups (HC, CD, and AB) and explored their correlation with alcohol‐related behavioral measures.

## 2 Methods

### 2.1 Design and Sample

This was a secondary analysis study investigating endocrine and immune biomarkers from a case–control study whose primary goal was to investigate gut microbial diversity and function in the context of AUD and whose main results were previously published [68]. A power analysis was conducted for the primary outcomes related to the microbiome, although recruitment for this study was stopped prematurely due to the COVID‐19 pandemic. The study included three groups: (1) healthy controls without AUD (HC, *N* = 12), (2) currently drinking, nontreatment seeking individuals with AUD (CD, *N* = 9), and (3) treatment‐seeking individuals with AUD who underwent a residential treatment program and were abstinent from alcohol for ≥ 6 weeks at the time of enrolment (AB, *N* = 10). The ≥ 6‐week abstinence time window of the AB group included at least 4 weeks in a residential treatment program at the NIH Clinical Center (Bethesda, Maryland), followed by at least 2 weeks of continued abstinence in the ‘real world’ after discharge from the inpatient unit. The mean abstinence duration was 61.08 ± 30.23 days with a range of 44–153 days. During the inpatient treatment program, participants in the AB group were provided standard of care, including therapy sessions and medications as needed. The CD group met criteria for AUD, similar to the AB group, and satisfied NIAAA criteria for heavy drinking (consumption of > 7 or 14 drinks/week for women or men, respectively [69]) but were not currently seeking treatment for AUD. The HC group had no current or past diagnosis of AUD, had to have low alcohol intake (≤ 1 drink/day for women and ≤ 2 drinks/day for men on average) and did not have any major neuropsychiatric disorders (see Data S1 and Data S2). HCs were enrolled to match sex, race, mean age (±5 years), and mean body mass index (BMI) of the two AUD groups (CD and AB). AUD diagnosis was determined by *Diagnostic and Statistical Manual of Mental Disorders 5th Edition* (*DSM‐5*) criteria [70] using the structured clinical interview for *DSM‐5* (SCID) [71].

Candidates for this study (17‐AA‐0093) were screened under the NIAAA screening and natural history protocol (14‐AA‐0181) and, if eligible, were enrolled after providing informed consent. A complete list of study inclusion and exclusion criteria can be found in the Supporting Information. The study included six outpatient visits at the NIH Clinical Center during which assessments were performed and samples were collected. Medical/psychiatric conditions reported by participants are listed in the Data S1 and Data S2 and summarized in Table S1.

Details on blood sample processing and ELISA can be found in the Supporting Information.

### 2.2 Data Analysis

For demographic and other screening measures (Table S1), a nonparametric Kruskal–Wallis test was conducted between the three groups with post hoc Dunn's multiple comparisons.

For the biomarker concentration data, normal distribution for each biomarker was evaluated in each group using a Shapiro–Wilk test. Nonnormally distributed data were transformed to improve normality. If a normal distribution was not achieved after transformation, a nonparametric test was conducted. For each biomarker, all information regarding normality, transformations, and result statistics can be found in Table 1. Values below the lower limit of quantification (LLOQ) were assigned 1/2 of the LLOQ [72]. This only occurred for two IL‐10 values (IL‐10 LLOQ = 0.38 pg/mL). Values that fell outside 2 standard deviations from the mean were removed from the analysis as outliers (1 value for Ghrelin in the AB group, 1 value for GH in the HC group, and 1 value for IL‐8 in the HC group).

**TABLE 1:** Normal distribution, transformation and group differences in biomarker data.

Biomarker	Normal distribution Y/N	Transformation	Statistics (main effect of group)
Immune biomarkers			
IL‐8	Y	N/A	*F*(2,29) = 6.33, *p* = 0.006^a^
IL‐18	Y	N/A	*F*(2,30) = 2.79, *p* = 0.08
CCL2	Y	N/A	*F*(2,30) = 3.20, *p* = 0.06
IL‐6	Y	N/A	*F*(2,30) = 2.07, *p* = 0.21
IL‐1RA	Y	N/A	*F*(2,30) = 0.41, *p* = 0.67
IL‐10	N	Quade nonparametric	*F*(2,27) = 1.60, *p* = 0.22
TNF‐α	N	Log^b^	*F*(2,30) = 2.78, *p* = 0.08
Endocrine biomarkers			
BDNF	N	Squared	*F*(2,30) = 4.34, *p* = 0.02^a^
GLP‐1	Y	N/A	*F*(2,25) = 4.22, *p* = 0.03^a^
GIP	Y	N/A	*F*(2,28) = 0.29, *p* = 0.75
Ghrelin	Y	N/A	*F*(2,27) = 3.02, *p* = 0.07
Insulin	N	Log^b^	*F*(2,30) = 0.36, *p* = 0.70
GH	N	Quade nonparametric	*F*(2,27) = 1.12, *p* = 0.34
Leptin	N	Log^b^	*F*(2,30) = 1.37, *p* = 0.27

Abbreviations: BDNF, brain‐derived neurotrophic factor; CCL2, chemokine ligand 2; GH, growth hormone; GIP, gastric inhibitory peptide; GLP‐1, glucagon‐like peptide 1; IL, interleukin; IL‐1ra, interleukin 1 receptor antagonist; TNF‐α, tumor necrosis factor 2.

^a^ *p* ≤ 0.05.

^b^ Log base 10.

To examine group differences in biomarker concentrations, one‐way ANCOVAs (or Quade nonparametric analysis of covariance for data that normal distribution could not be achieved) were conducted with group (HC, CD, and AB) as the between‐subjects factor, and sex and age as covariates. Sex and age were used as covariates because many of the biomarkers showed a main effect of sex (IL‐8, GLP‐1, and leptin) and/or age (IL‐8, CCL2, IL‐6, and GLP‐1), and many of these biomarkers have previously been shown to be sensitive to sex and age [73, 74, 75, 76, 77, 78, 79, 80]. Post hoc Bonferroni tests were conducted where there was a main effect of group. Effect sizes are reported as partial eta squared (ƞ_p_^2^) for biomarkers showing a main effect of group. All graphs show group mean ± standard deviation. Statistical significance was set at *p* ≤ 0.05, two‐tailed.

In addition to the main analyses, we conducted a principal component analysis (PCA) for the proinflammatory (IL‐8, IL‐18, IL‐6, CCL2, and TNF‐α) and anti‐inflammatory (IL‐1RA and IL‐10) biomarkers, separately, to create an aggregate proinflammatory and anti‐inflammatory value, respectively. Details related to the PCA can be found in the Supporting Information.

We previously reported some differences in baseline behavioral measures between the two AUD groups (CD vs. AB), which were collected prior to abstinence in the AB group [68]. Here, we conducted exploratory correlational analyses between these behavioral measures (alcohol use, AUD severity, and anxiety/depression) and the immune and endocrine biomarker concentrations. Spearman's *r* was calculated for all correlations due to many of the outcome variables not being normally distributed. Behavioral measures used in this analysis included average number of standard drinks [81] per day and number of heavy drinking days according to the 90‐day alcohol timeline follow back (aTLFB) [82], the Alcohol Use Disorder Identification Test (AUDIT, [83]), the Alcohol Dependence Scale (ADS, [84]), the Obsessive Compulsive Drinking Scale (OCDS, [85]), the Brief Scale for Anxiety (BSA, [86]), the State‐Trait Anxiety Inventory–Trait (STAI‐T, [87]) and the Montgomery–Asberg Depression Rating Scale (MADRS, [88]). Note that AUD severity in this context does not refer to the *DSM‐5* categories of mild, moderate, and severe AUD based on the number of criteria endorsed. We use the term here and throughout this publication in reference to the burden or extent of disease for AUD based on the following measures: AUDIT, ADS, and OCDS.

IBM SPSS (Version 28.0 for Windows, IBM Corp., Armonk, New York, USA), R (Version 4.1.1, ggplot and reshape2 packages were used), and Prism (Version 10.0.0 for Windows, GraphPad Software, Boston, Massachusetts, USA) software were used for data analyses and visualisation.

## 3 Results

There were no differences in demographics between the three group, including sex, race, ethnicity, age, and BMI. There were group differences in other AUD severity and anxiety/depression measures and medical conditions as previously reported [68] (Table S1).

Overall results of group difference analyses for biomarkers data are presented in Table 1 and further explained below.

### 3.1 Group Differences in Circulating Immune Markers (Figure 1)

**FIGURE 1:**
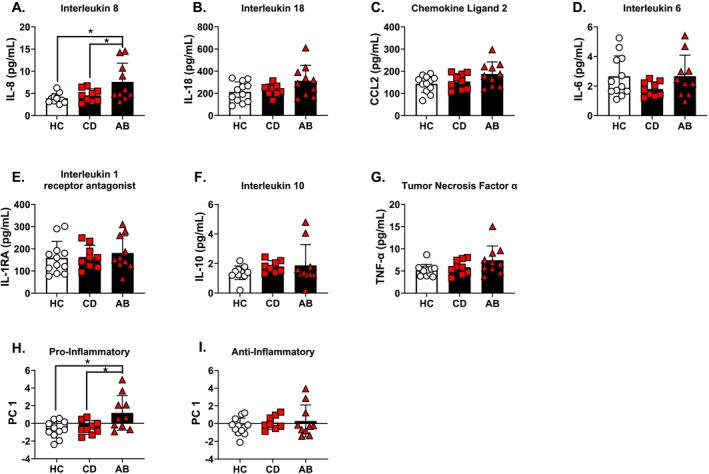
Circulating immune markers across the three groups. (A) IL‐8 was elevated in the AB group compared to CD and HC. (B) IL‐18 showed a trend for a main effect of group. (C) CCL2 showed a trend for a main effect of group. (D) IL‐6 did not differ between groups. (E) IL‐1ra did not differ between groups. (F) IL‐10 did not differ between groups. (G) TNF‐α concentrations were log transformed and showed a trend for a main effect of group. (H) Proinflammatory aggregate PC1 values were elevated in the AB group compared to CD and HC. (I) Anti‐inflammatory aggregate PC1 values did not show groups differences. Untransformed values (mean ± standard deviation) are displayed in the graph. *p ≤ 0.05.

Immune biomarkers showed a group effect for IL‐8 (Figure 1A) (*F*(2,29) = 6.33, *p* = 0.006, ƞ_p_^2^ = 0.318) with AB > CD (*p* = 0.03) and AB > HC (*p* = 0.008). There was a trend for a group effect for IL‐18 (Figure 1B) (*F*(2,30) = 2.79, *p* = 0.08, ƞ_p_^2^ = 0.177) and CCL2 (Figure 1C) (*F*(2,30) = 3.20, *p* = 0.06, ƞ_p_^2^ = 0.197) concentrations. IL‐6 (Figure 1D) and IL‐1RA (Figure 1E) were not different between the groups. No effect of group was found for IL‐10 (Figure 1F). No effect of group was found for TNF‐α (Figure 1G). The proinflammatory PC1 data (Figure 1H) showed a main effect of group (*F*(2,29) = 6.98, *p* = 0.004) with the AB group showing higher proinflammatory PC1 values compared to both CD (*p* = 0.02) and HC (*p* = 0.007). The anti‐inflammatory PC1 values showed no group differences (Figure 1I). Overall, these findings indicate a proinflammatory state in the AB group, but not in the CD group, compared to HCs.

### 3.2 Group Differences in Circulating Endocrine Markers (Figure 2)

**FIGURE 2:**
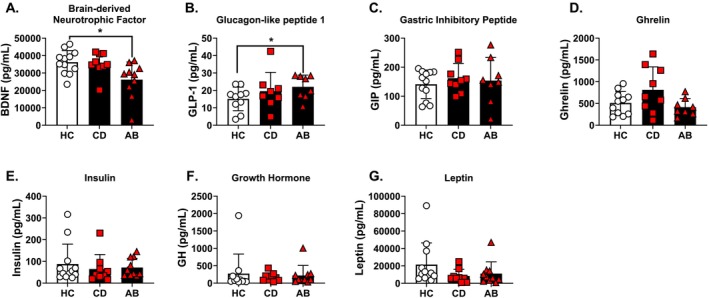
Circulating endocrine markers across the three groups. (A) BDNF values were squared to achieve a normal distribution. BDNF concentrations decreased in the AB group compared to HC. (B) GLP‐1 was elevated in AB compared to HC. (C) GIP concentrations did not differ between groups. (D) Ghrelin concentrations were trending towards a main effect of group. (E) Insulin values were log transformed and did not differ between groups. (F) Growth hormone did not differ between groups. (G) Leptin was log transformed and did not differ between groups. Untransformed values (mean ± standard deviation) are displayed in the graph. *p ≤ 0.05.

For BDNF data (Figure 2A), there was a main effect of group (*F*(2,26) = 4.34, *p* = 0.02, ƞ_p_^2^ = 0.266) with AB > HC (*p* = 0.04). There was a main effect of group for GLP‐1 (Figure 2B) (*F*(2,25) = 4.22, *p* = 0.03, ƞ_p_^2^ = 0.287) with AB > HC (*p* = 0.03). GIP concentrations (Figure 2C) did not differ between groups. Ghrelin concentrations (Figure 2D) were trending towards a main effect of group (*F*(2,27) = 3.02, *p* = 0.07, ƞ_p_^2^ = 0.208). Insulin (Figure 2E) data showed no effect of group. GH (Figure 2F) data showed no effect of group. Leptin (Figure 2G) data showed no effect of group. Overall, these findings indicate differences in circulating BDNF and GLP‐1 concentrations in the AB group, but not in the CD group, compared to HCs.

### 3.3 Correlations Between Protein Concentrations and Behavioral Measures (Figure 3)

**FIGURE 3:**
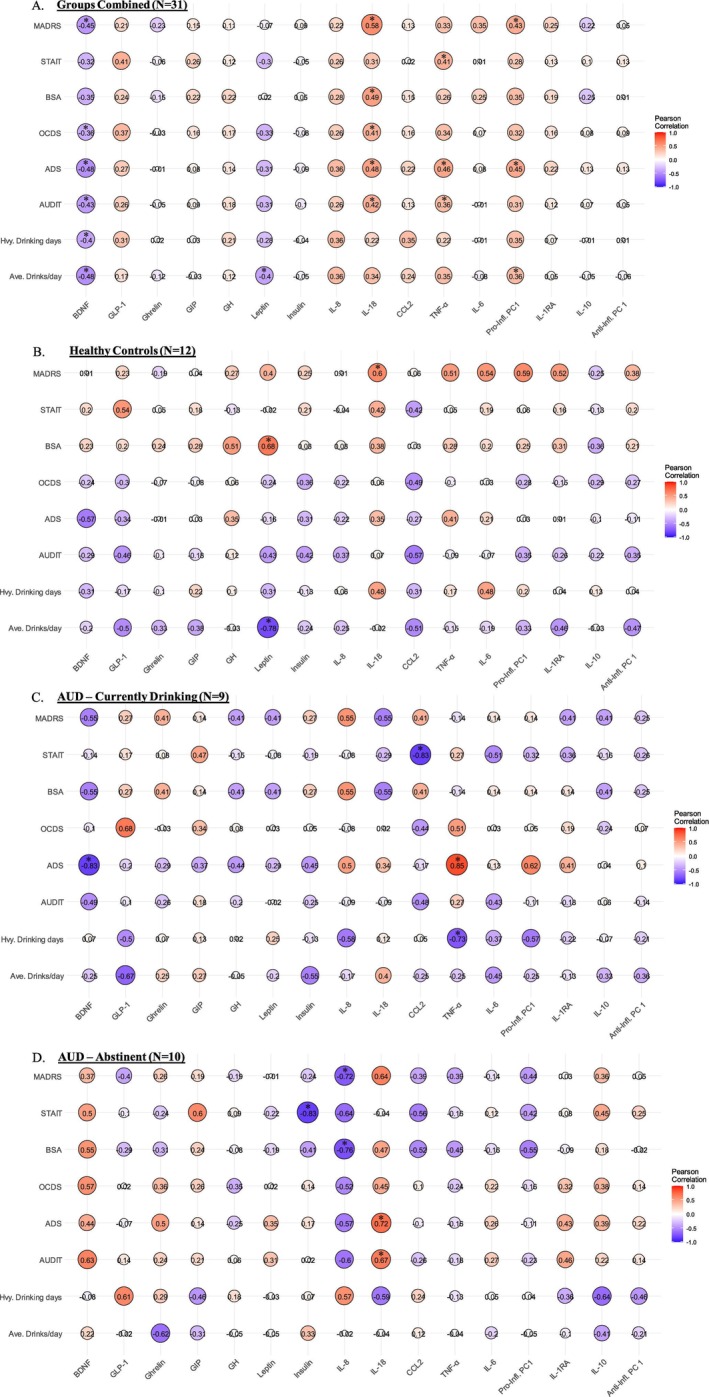
Correlations between protein concentrations and behavioral measures. (A) Combined sample, (B) healthy controls (HC), (C) AUD—currently drinking (CD), and (D) AUD—abstinent (AB). Spearman's *r* values are shown in the matrix graphs. Circle size and color (red—positive correlation, blue—negative correlation) are proportional to the magnitude of the correlation. *MADRS*—Montgomery–Asberg Depression Rating Scale. *STAIT*—State‐Trait Anxiety Inventory–Trait. *BSA*—Brief Scale for Anxiety. *OCDS*—Obsessive Compulsive Drinking Scale. *ADS*—Alcohol Dependence Scale. AUDIT—Alcohol Use Disorder Identification Test. *Hvy. drinking Days*—90‐day alcohol timeline follow back (aTLFB) number of heavy drinking days. *Ave. drinks/day*—90‐day alcohol timeline follow back (aTLFB) average drinks per day. *p ≤ 0.05.

Baseline behavioral measures of alcohol consumption, AUD severity and depression/anxiety were tested for correlations with all reported biomarkers, both in the combined sample (*N* = 31) and within each group separately (HC, CD, AB). Spearman's *r* values are displayed in the correlation matrices in Figure 3. Of note, the combined analysis (Figure 3A) showed BDNF to be negatively correlated with past drinking, AUDIT, ADS, OCDS and MADRS. IL‐18 positively correlated with AUDIT, ADS, OCDS, BSA and anxiety/depression measures (Figure 3A), and TNF‐α positively correlated with ADS, AUDIT and STAI‐T (Figure 3A). The proinflammatory aggregate PC1 values positively correlated with aTLFB average drinks per day, ADS and MADRS in combined groups (Figure 3A). In the within‐group analyses, among the CDs only (Figure 3C), BDNF negatively correlated with ADS; TNF‐α positively correlated with ADS but negatively correlated with past heavy drinking days. In the AB group only (Figure 3D), IL‐8 negatively correlated with BSA and MADRS, whereas IL‐18 positively correlated with AUDIT and ADS. Overall, these results indicate associations between several of the biomarkers that showed group differences and baseline measures of alcohol drinking, AUD severity, and anxiety/depression.

## 4 Discussion

These results demonstrate elevated IL‐8 and GLP‐1, as well as decreased BDNF, after controlling for sex and age in a group of people with AUD who remained abstinent for at least 6 weeks (AB), compared to a matched HC group (Figures 1 and 2). These changes were not observed when comparing a group of nontreatment‐seeking and currently drinking people with AUD (CD) to the same HC group. It is important to note that the AB group reported differences in baseline behavioral measures and medical conditions, collected prior to abstinence, compared to HC and CD (Table S1 and [68]). Therefore, exploratory correlational analyses were conducted between selected behavioral measures and biomarkers to better understand the correlates of the observed differences. In the combined sample (Figure 3A), BDNF negatively correlated with past drinking (aTLFB), AUD severity, and depression measures. IL‐18, TNF‐α, and the proinflammatory PC1 values positively correlated with AUD severity and anxiety/depression measures. All data presented here demonstrate changes in circulating immune and endocrine proteins in people with AUD who have remained abstinent for 6+ weeks compared to HC. Furthermore, differences were mostly observed in the AUD—abstinent group (AB) rather than the AUD—currently drinking (CD) group. These differences likely reflect group differences in baseline preabstinence measures of drinking levels, AUD severity, and anxiety/depression measures rather than abstinence from alcohol because the biomarker changes correlated with these baseline assessments and concentrations were stable over the course of the six study sessions. However, a limitation of this study is that we do not have blood protein data collected preabstinence for the AB group to determine how biomarkers may have changed over the course of abstinence. These findings build on our previous work that showed gut microbiome diversity and metabolome were different in the AB group compared to both CD and HC [68]. These findings are likely driven by the fact that the AB group has a different phenotypic profile as compared to the CD group, for example in terms not only of more severe AUD but also of more health comorbidities and complications (see Data S1 and Data S2 and Table S1).

IL‐8 concentrations were elevated in the AB group compared to CD and HC groups, and other proinflammatory markers showed a trend in the same direction. The proinflammatory PC1 values were elevated in the AB group compared to both CD and HC groups. These data suggest a proinflammatory state in the AB group. IL‐8 is a proinflammatory chemokine that primarily functions to recruit and activate neutrophils during inflammatory responses and is associated with various diseases involving chronic inflammation and immune dysregulation [89]. However, we did not observe group differences in neutrophils or most CBC measures, but we did observe an increase in mean platelet volume (MPV) in the AB group relative to HC and CD (Figure S3). The increase in MPV may indicate an inflammatory state in the AB group, which is corroborated by the protein biomarker data. While we did not observe group difference in WBC, neutrophils have been shown to be elevated in people with a family history of AUD/SUD, indicting this marker as a potential vulnerability factor [90]. Acute alcohol use also affects IL‐8 concentrations. In moderate drinkers, IL‐8 was elevated 6 h post alcohol consumption and returned to baseline concentrations 24 h post alcohol consumption, demonstrating that alcohol acutely and transiently elevates IL‐8 [91]. However, other studies have found that acute alcohol consumption attenuates IL‐8 concentrations 4 h post consumption [92]. Therefore, it is likely that alcohol affects IL‐8 concentrations in a dynamic fashion that may differ based on alcohol dose and time of sample collection. These acute increases in IL‐8 may be due to increases in intestinal permeability by alcohol that results in gut microbiome and metabolome translocation and activation of an inflammatory response [93]. In addition to acute alcohol effects, differential concentrations of IL‐8 have been found in people with AUD. One study found that IL‐8 concentrations were elevated at the start of an alcohol detoxification program (early withdrawal) but returned close to values observed in healthy controls 4 weeks into the program [94]. Additionally, another study found increased concentrations of proinflammatory markers at the start of an alcohol detoxification program that were largely reduced to control concentrations following 3 weeks of abstinence [13]. These data would appear to be counter to the present findings showing elevated IL‐8 in the abstinent AUD group but not in the currently drinking AUD group. However, these findings support the conclusion that group differences observed in the present study likely reflect the chronicity and severity of AUD rather than short‐term abstinence per se. It is possible that IL‐8 concentrations were higher in the AB group at the start of alcohol abstinence and remained elevated due to the high severity of AUD in this group. The present study also found a trend for elevated IL‐18 concentrations in the AB group. IL‐18 is a proinflammatory cytokine that plays a key role in promoting immune responses by inducing interferon‐gamma (IFN‐γ) production, and it is implicated in inflammation‐driven diseases, including autoimmune disorders, metabolic syndromes, and chronic inflammatory conditions [95]. One study found that IL‐18 mRNA levels were elevated in unstimulated PBMCs from participants with severe alcohol‐induced liver cirrhosis compared to healthy controls [96]. IL‐18 signalling was disrupted in the central amygdala (a key brain hub related to addiction and stress) of a rat model of AUD and comorbid PTSD [97]. Furthermore, IL‐18 gene expression was upregulated in postmortem brain tissue in people with AUD compared to healthy controls. In summary, our findings further implicate IL‐8 and IL‐18 as important markers closely associated with AUD. Interestingly, in the combined sample, IL‐18, TNF‐α, and the proinflammatory PC‐1 values positively correlated with measures of AUD severity and anxiety/depression measures. In the AB group alone, IL‐8 negatively correlated with anxiety/depression measures, and IL‐18 positively correlated with AUD severity measures. Therefore, proinflammatory biomarkers were associated with AUD during abstinence in the present study. However, other longitudinal studies have shown that the inflammatory effects observed in people with AUD are attenuated after a period of alcohol abstinence [13]. This again suggests that the changes observed in the AB group may have been more pronounced during the start of abstinence.

In addition to the immune changes, BDNF concentrations were decreased in the AB group compared to the HC group. BDNF is a neurotrophic factor involved in synaptic plasticity and neurogenesis [98]. BDNF also has peripheral effects on the pancreas [44] and other organs and is involved in the neuroendocrine regulation of feeding [43]. Many studies have examined BDNF concentrations in people with AUD, several of which find that AUD is associated with decreased circulating BDNF concentrations [32, 34, 35, 36, 37]. Another study found that BDNF concentrations negatively correlated with average drinks per drinking day [33]. Additionally, decreased BDNF concentrations are associated with other neuropsychiatric conditions like depression and schizophrenia [99, 100, 101]. A meta‐analysis suggests that BDNF concentrations may increase over time during periods of abstinence from alcohol [32]. These findings appear counter to the results of the present findings showing decreased concentrations of BDNF in abstinent people with AUD. However, again, differences in AUD severity between abstinent and currently drinking groups may explain this discrepancy. In people with severe AUD, deficits in BDNF may be sustained for relatively long periods of time during abstinence. In the full sample, BDNF was negatively correlated with history of alcohol intake, AUD severity, and depression measures, further implicating these preabstinent group differences as the primary source of group differences in biomarkers. These findings corroborate previous results from our team showing that BDNF concentrations negatively correlated with OCDS scores in people with AUD [33], as well as a wealth of literature implicating deficits in BDNF in a multitude of neuropsychiatric conditions [32, 101]. As such, interventions that directly or indirectly boost BDNF concentrations may have therapeutic benefits for people with AUD.

The neuroendocrine peptide GLP‐1 was also elevated in the AB group compared to HC. Previous studies from our lab have shown that acute oral and intravenous administration of alcohol decreases circulating concentrations of GLP‐1 [51], and this has been further demonstrated in a recent study with a rigorous within‐subject design where alcohol administration was compared to placebo [102]. As such, the AB group may show higher GLP‐1 concentrations in the present study as a compensatory mechanism from previous heavy alcohol use. Furthermore, our team and others have shown that GLP‐1 receptor agonists reduce alcohol intake in preclinical models and could be promising medications for AUD [23]. While GLP‐1‐based medications have revolutionized the management of Type 2 diabetes mellitus and obesity, results from ongoing clinical trials are needed to evaluate their potential efficacy in the treatment of AUD [27, 103].

The present study has limitations. The small sample size for each group (*N* ~ 10 per group) decreases statistical power. The risk of collecting a biased/unrepresentative sample is increased, which could increase risk of false‐positive/negative results and limits the generalizability of the findings. Data collection for this study was stopped prematurely due to the COVID‐19 pandemic. As such, these results may be better interpreted as hypothesis‐generating and need confirmation with larger sample sizes. Another limitation was that the two AUD groups differed in drinking, AUD severity and anxiety/depression measures prior to the AB group starting abstinence. This is not surprising as treatment‐seeking individuals tend to have more severe AUD and comorbidities than nontreatment‐seeking participants [104, 105, 106, 107]. Nevertheless, this difference complicates conclusions about whether the observed differences in immune and endocrine markers were driven by abstinence from alcohol versus preabstinence measures of drinking history, AUD severity, and anxiety/depression measures. As such, the main findings from this study can be best conceptualized as two separate comparisons: (1) between an abstinent group (at least 6 weeks) of treatment‐seeking individuals who had heavy alcohol use and severe AUD at the time of starting inpatient treatment and a matched HC group and (2) between a nontreatment‐seeking, currently drinking group with AUD and a matched HC group. The negative findings between the AUD—currently drinking group and HC group may be the result of a Type 2 error (false negative) given the low sample size. Other studies have shown changes in these biomarkers in currently drinking people with AUD [108]. Another potential limitation is relying on self‐reported measures of alcohol use [109]. For example, we cannot be completely certain that the AB group did not consume any alcohol between the time of discharge from the inpatient unit to the time of enrolment into this study and blood sample collection. However, in addition to the aTLFB indicating total alcohol abstinence, breathalyzers were conducted prior to sample collection of each study visit, and all participants showed a negative breathalyzer for all six study visits. Furthermore, the ABs were a group of highly motivated, treatment‐seeking individuals who were able to maintain at least 4 weeks of alcohol abstinence during their inpatient stay. Together, these factors make the possibility that the AB group drank alcohol between the time of inpatient discharge and biospecimen sample collection less likely, although we acknowledge that future studies like this should also include biomarker confirmation of abstinence such as PEth. The lack of IL‐1β while IL‐1RA was included in the present analysis leaves an incomplete picture of IL‐1 receptor signalling given that IL‐1β is an agonist and IL‐1RA is an antagonist, of IL‐1 receptor, and have pro‐ and anti‐inflammatory effects, respectively [110]. Finally, while we conducted aggregate values using PC analysis for pro‐ and anti‐inflammatory biomarkers to provide a general overview of the pro‐ versus anti‐inflammatory state of the groups, these data should be interpreted with caution as some biomarkers can have both pro‐ or anti‐inflammatory effects depending on the biological context [111, 112].

Together, the present study demonstrated changes in circulating IL‐8, GLP‐1, and BDNF concentrations in a group of abstinent individuals with a history of heavy alcohol use and severe AUD compared to a matched group of healthy controls. It could be speculated that these changes are related as GLP‐1R receptor agonists and BDNF have shown to have anti‐inflammatory effects [58, 59]. In fact, correlational analyses in combined groups between the immune and endocrine biomarkers showed negative associations between BDNF versus TNF‐α and the proinflammatory PC1 values. GLP‐1 concentrations positively correlated with IL‐1RA and the anti‐inflammatory PC1 values. In contrast to findings in the AB group, individuals with less severe AUD, who were still drinking and had fewer associated complications, demonstrated nearly no changes in circulating immune or endocrine proteins compared to healthy controls. While more work is needed, these data provide novel insight into immune and endocrine correlates of alcohol use, many of which are being studied, or at least considered, as potential therapeutic targets for AUD.

## Conflicts of Interest

Outside his NIH work, Dr. Lorenzo Leggio receives an honorarium from UK Medical Council on Alcohol as Editor‐in‐Chief for Alcohol and Alcoholism and he also receives royalties from Rutledge as co‐editor of a textbook. The other authors declare no competing interests.

## Supporting information


**Table S1** Participant demographics and characteristics.
**Table S2** Assays for all proteins tested.
**Table S3** Correlations between immune and endocrine biomarkers.
**Figure S1** Immune proteins over the course of the six study visits.
**Figure S2** Endocrine proteins over the course of the six study visits.
**Figure S3** Complete blood count (CBC).

**Data S1.** Raw data values.

**Data S2.** Medical conditions.

## Data Availability

The data that support the findings of this study are available from the corresponding author upon reasonable request.
