# Supplementary material for: Circulating Immune and Endocrine Markers in Currently Drinking and Abstinent Individuals With Alcohol Use Disorder and Controls
Source: Addict Biol. 2025 May 2;30(5):e70039. doi: 10.1111/adb.70039 (PMC12046569; doi:10.1111/adb.70039)
Supplement: Supplementary file 1 — Table S1 Participant demographics and characteristics. Table S2 Assays for all proteins tested. Table S3 Correlations between immune and endocrine biomarkers. Figure S1 Immune proteins over the course of the six study visits. Figure S2 Endocrine proteins over the course of the six study visits. Figure S3 Complete blood count (CBC). [file ADB-30-e70039-s002.docx]

**SUPPLEMENTAL MATERIAL**

Circulating immune and endocrine markers in currently drinking and abstinent individuals with alcohol use disorder and controls

Ryan E. Tyler^1,2^, Carlotta Vizioli^3^, Jennifer J. Barb^4^, Mehdi Farokhnia^1*^, Lorenzo Leggio^1*^

^1^ Clinical Psychoneuroendocrinology and Neuropsychopharmacology (CPN) Section, Translational Addiction Medicine Branch, National Institute on Drug Abuse Intramural Research Program and National Institute on Alcohol Abuse and Alcoholism Division of Intramural Clinical and Biological Research, National Institutes of Health, Baltimore, MD, USA.

^2^ National Institute of General Medical Sciences, 45 Center Drive, Bethesda, MD 20892, USA

^3^ Interoceptive Disorders Unit, Office of the Clinical Director, National Institute of Neurological Disorders and Stroke, NIH, Bethesda, MD, USA.

^4^ Translational Biobehavioral and Health Disparities Branch, Clinical Center, NIH, Bethesda, MD, USA.

**SUPPLEMENTAL METHODS**

***Study Inclusion Criteria***

*All Participants*

- Male or female individuals 21-70 years old (inclusive)

*Specific for Abstinent Group: AB*

- Current Alcohol Use Disorder (AUD) by DSM-5 criteria
- Being alcohol abstinent for at least 4 weeks with minimum of 2 weeks in a non-protective environment at the time of study screening

*Specific for Current Drinking: CD*

- Current Alcohol Use Disorder (AUD) by DSM-5 criteria
- Non-treatment seeking for alcohol use

Satisfying heavy drinking criteria during 4-weeks prior to screening “for men, >14 standard drinks in any one week **and** ≥4 drinks per occasion at least once per month over the past 30 days; for women, >7 drinks per week **and** ≥3 drinks per occasion at least once per month over the past 30 days” **and** any drinking during the 2-day prior to signing the study-specific consent.

*Specific for Healthy Controls: HC*

- No current or past diagnostic of AUD by DSM-5 criteria
- Non-alcohol drinkers or moderate alcohol drinkers i.e., up to 1 drink per day on average and not meeting NIAAA criteria for:
- heavy drinking (i.e., for men, >14 standard drinks in any one week or ≥4 drinks per occasion at least once per month over the past 30 days; for women, >7 drinks per week or ≥3 drinks per occasion at least at least once per month over the past 30 days)
- or binge drinking (i.e., drinking 5 or more standard drinks on the same occasion on at least 1 day in the past 30 days for both male and female)

***Study Exclusion Criteria***

- Current pregnancy or lactation.

- Positive urine drug test for illegal drugs.

- Presence of active implantable electronic devices (e.g., defibrillators, pumps, pacemakers).

- The following current medical conditions: diabetes; chronic gut inflammatory diseases; gastrointestinal or any other type of cancer; short bowel syndrome; conditions requiring parenteral nutrition.

- Self-reported diarrhea or other symptoms of possible enteritis (past 7 days).

- Recent history of sigmoidoscopy or colonoscopy (past 30 days).

- Current use (past 90 days) of the following medications: oral and/or intravenous (IV) antimicrobials (specifically: antiviral, antifungal, or antibiotics); prebiotics; probiotics; laxatives; antispasmodic drugs; oral, intramuscular (IM) or IV steroids

- Any other reason or clinical condition that the Principal Investigator (PI) or Medical Advisory Investigator (MAI) considers unsafe for the individual or not in the best interest of the study research integrity.

**Blood Sample Collection and Processing**

Bloods samples were collected on the first day of the study prior to any major assessments. All blood samples were collected at approximately 10am prior to eating breakfast and participants had no food or water for at least 2 hours before sample collection. Blood samples over the course of the six study visits were also collected and are shown in Supplemental Fig. S1 and S2. Group difference analyses were performed on the first sample collected as biomarker concentrations were stable across the six study visits.

Venous blood samples were collected to isolate both plasma and serum samples from whole blood. Plasma samples were collected in LAV3.0 Light Lavender tubes 3mL K2EDTA 5.4 mg (LVV, BD Vacutainer^®^, Becton, Dickinson and Company, Franklin Lakes, NJ) and serum samples were collected in Serum Separator Tubes (SST) 4 mL (VACUETTE^®^, Greiner Bio-One International GmbH, Kremsmünster, Austria). Plasma samples were spun within 30-min of collection in a centrifuge (15-min, 1700 g, 4º C). Serum samples incubated at room temperature for at least 30-min before spinning (10-min, 1000 g, 25º C). After spinning, the top-layer plasma or serum supernatant was isolated, and aliquots were stored at -80°C before enzyme-linked immunosorbent assay (ELISA) testing. Proteins measured included ghrelin (“acyl-ghrelin”), GIP, total GLP-1, and all immune proteins: Interleukin 8 (IL-8), IL-18, IL-6, chemokine ligand 2 (CCL2), and tumor necrosis factor α (TNF-α), IL-10, and IL-1 receptor antagonist (IL-1RA). Serum samples were collected to measure BDNF, GH, leptin, and insulin.

ELISA assays were used to measure concentrations of specific analytes. Some groups of analytes were measured using a multi-analyte ELISA (ELLA). All assays were run following the manufacturers’ instructions. A Spectrophotometer (ClarioStar Plus, BMG Labtech, Ortenburg, Germany) was used to take absorbance measurements. A separate standard curve (absorbance *vs*. concentration) was collected for each 96-well plate to calculate protein concentrations in pg/mL. Table S1 shows blood sample collection and ELISA information for each analyte.

**Supplemental Data Analysis**

Principal component analysis was conducted as a data reduction method using the pro-inflammatory biomarkers and the anti-inflammatory biomarkers separately as a way to generate aggregate pro- and anti-inflammatory values. For the pro-inflammatory biomarkers, the first principal component (PC1) explained 47.96% of the total variance. For the anti-inflammatory biomarkers, PC1 explained 78.18% of the total variance. Loadings (relative weights) are displayed in the table below.

| Biomarker | Loading |
| --- | --- |
| Pro-inflammatory | |
| IL-8 | 0.826 |
| IL-18 | 0.306 |
| CCL2 | 0.792 |
| TNF-α | 0.855 |
| IL-6 | 0.515 |
| Anti-inflammatory | |
| IL-1RA | 0.884 |
| IL-10 | 0.884 |

**SUPPLEMENTAL TABLES**

**Table S1 – Participant Demographics and Characteristics**

| **Variables** | **Group** | | |
| --- | --- | --- | --- |
|  |  |  |  |
| ***Demographic*** | **HC** | **CD** | **AB** |
| Sex |  |  |  |
| Female (N) | 4 | 2 | 2 |
| Male (N) | 8 | 7 | 8 |
| Race |  |  |  |
| White/Caucasian (N) | 7 | 7 | 6 |
| Black/African American (N) | 5 | 2 | 3 |
| Asian (N) | 0 | 0 | 1 |
| Ethnicity |  |  |  |
| Hispanic/Latino (N) | 1 | 0 | 0 |
| Non-Hispanic/Latino (N) | 11 | 9 | 10 |
| Age, Years | 48.8 (12.0) | 45 (12.6) | 45.9 (11.4) |
| BMI, kg/m^2^ | 28.8 (4.9) | 25.7 (4.0) | 26.7 (5.1) |
| Smoker (N) | 0 | 4 | 6 |
|  |  |  |  |
| ***Alcohol-related*** |  |  |  |
| AUDIT | 2 (1.6) | 16 (5.6)* | 28 (7.7)* |
| ADS | 0 | 10 (6.1)* | 22 (9.2)* |
| OCDS | 1.3 (1.5) | 12.3 (3.7)* | 22.1 (8.8)* |
| Heavy drinking years - lifetime history | 0 | 12.1 (13.8) | 16.4 (9.4)* |
| 90-d aTLFB average drinks per day | 0.3 (0.3) | 3.7 (1.2)* | 13.6 (8)* |
| 90-d aTLFB heavy drinking days | 13.6 (14.3) | 66.0 (20.8)* | 72.6 (23.6)* |
| Days of abstinence from alcohol | N/A | N/A | 61.08 (30.23) |
|  |  |  |  |
| ***Psychological*** |  |  |  |
| MADRS | 1.3 (1.7) | 0.38 (1.1) | 15 (7.8)*^#^ |
| BSA | 1.2 (1.7) | 0.11 (0.3) | 11 (6.6) *^#^ |
| STAI-T | 24.8 (4.2) | 35.6 (7.7) | 48.1 (8.7)* |
|  |  |  |  |
| Medical Conditions | 1.58 (1.3) | 1.33 (1.2) | 4.0 (1.8) *^#^ |

**AUDIT - Alcohol Use Disorder Identification Test. Hvy. OCDS - Obsessive Compulsive Drinking Scale. ADS - Alcohol Dependence Scale. Drinking Days - 90-day alcohol timeline follow back (aTLFB) number of heavy drinking days. Ave. Drinks/day - 90-day alcohol timeline follow back (aTLFB) average drinks per day. MADRS – Montgomery-Asberg Depression Rating Scale. BSA - Brief Scale for Anxiety. STAIT - State-Trait Anxiety Inventory – Trait. Kruskal-Wallis tests and Dunn’s multiple comparisons used for all analyses. * significant difference compared to HC (p < 0.05). # significant difference compared to CD (p < 0.05). HC = Healthy Controls; CD = AUD – Currently Drinking; AB = AUD – Abstinent.**

**Data are shown as either total number (N) or group mean ± std. deviation. See also: Piacentino D, Vizioli C, Barb JJ, Grant-Beurmann S, Bouhlal S, Battista JT, Jennings O, Lee MR, Schwandt ML, Walter P, Henderson WA, Chen K, Turner S, Yang S, Fraser CM, Farinelli LA, Farokhnia M, Leggio L. Gut microbial diversity and functional characterization in people with alcohol use disorder: A case-control study. PLoS One 2024; 19(6):e0302195**

**Table S2 – Assays for all proteins tested.**

| Analyte | Blood Type | Tube collection type | Blood collection tube treatment | Assay, Dilution Factor |
| --- | --- | --- | --- | --- |
| Ghrelin  (Acyl-Ghrelin) | Plasma | LVV | 0.1M Pefabloc* SC, 5% HCl (v/v) | Millipore EZGRA-88K, Neat |
| GLP-1  (Total) | Plasma | LVV | DPP-IV** (10uL/mL blood) | Millipore EZGLP1T-36K, Neat |
| GIP | Plasma | LVV | DPP-IV (10uL/mL blood) | RayBiotech EIA-GIP-1, 1:4 |
| BDNF, Growth Hormone, Insulin, Leptin | Serum | SST | None | Proteinsimple  SPCKC-P3-004003, 1:2 |
| Immune Biomarkers*** | Plasma | LVV | DPP-IV (10uL/mL blood) | Proteinsimple  SPCKE-P3-003746, 1:2 |

*Peflabloc – Pefabloc SC AEBSF - 4-(2-aminoethyl)benzenesulfonyl fluoride hydrochloride (Roche Diagnostics GmbH, Germany – Pefabloc^®^ SC)

**DPP-IV – dipeptidyl peptidase IV inhibitor (EMD Millipore Corp., Billerica, MA – Cat. #DPP4-010)

***Immune biomarkers - IL-8, IL-18, CCL2, TNF-α, IL-1RA, IL-6, and IL-10

**Table S3 – Correlations between immune and endocrine biomarkers**

**A.**

| **Groups Combined (N=31)** | **BDNF** | **GLP-1** | **Ghrelin** | **GIP** | **GH** | **Leptin** | **Insulin** |
| --- | --- | --- | --- | --- | --- | --- | --- |
| **IL-8** | -0.32 | 0.31 | 0.04 | 0.04 | 0.10 | -0.32 | 0.18 |
| **IL-18** | -0.19 | 0.16 | -0.02 | 0.18 | 0.16 | -0.02 | 0.15 |
| **CCL2** | -0.15 | 0.21 | -0.29 | -0.15 | -0.17 | -0.10 | 0.37 |
| **TNF-α** | -0.53* | 0.28 | -0.11 | 0.00 | 0.02 | -0.25 | 0.12 |
| **IL-6** | -0.02 | 0.49* | -0.20 | 0.18 | -0.05 | 0.18 | 0.33 |
| **Pro-Infl. PC 1** | -0.38* | 0.34 | -0.17 | -0.03 | -0.12 | -0.11 | 0.37* |
| **IL-1RA** | -0.16 | 0.61* | -0.18 | 0.34 | -0.13 | 0.26 | 0.35 |
| **IL-10** | 0.05 | 0.20 | -0.04 | 0.17 | -0.30 | -0.03 | 0.15 |
| **Anti-Infl. PC 1** | -0.01 | 0.56* | -0.20 | 0.41* | -0.21 | 0.25 | 0.40* |

**B.**

| **Healthy Controls (N=12)** | **BDNF** | **GLP-1** | **Ghrelin** | **GIP** | **GH** | **Leptin** | **Insulin** |
| --- | --- | --- | --- | --- | --- | --- | --- |
| **IL-8** | 0.66* | 0.43 | -0.33 | 0.37 | -0.25 | 0.36 | 0.59 |
| **IL-18** | -0.06 | 0.30 | -0.05 | 0.36 | 0.28 | 0.10 | 0.35 |
| **CCL2** | 0.18 | 0.43 | -0.19 | -0.23 | -0.27 | 0.47 | 0.43 |
| **TNF-α** | -0.40 | 0.12 | -0.07 | -0.25 | 0.44 | 0.13 | 0.22 |
| **IL-6** | 0.13 | 0.46 | -0.33 | 0.28 | -0.31 | 0.30 | 0.53 |
| **Pro-Infl. PC 1** | 0.19 | 0.58 | -0.54 | 0.08 | -0.19 | 0.35 | 0.77* |
| **IL-1RA** | 0.01 | 0.68* | -0.41 | 0.50 | -0.45 | 0.55 | 0.62* |
| **IL-10** | 0.06 | 0.20 | -0.13 | 0.02 | -0.61 | -0.23 | 0.29 |
| **Anti-Infl. PC 1** | 0.16 | 0.72* | -0.30 | 0.54 | -0.55 | 0.50 | 0.67* |

**C.**

| **AUD – Currently Drinking (N=9)** | **BDNF** | **GLP-1** | **Ghrelin** | **GIP** | **GH** | **Leptin** | **Insulin** |
| --- | --- | --- | --- | --- | --- | --- | --- |
| **IL-8** | -0.48 | -0.21 | 0.30 | -0.23 | -0.02 | -0.77* | -0.43 |
| **IL-18** | -0.63 | -0.17 | 0.45 | -0.18 | 0.22 | -0.38 | -0.35 |
| **CCL2** | 0.22 | -0.17 | -0.17 | -0.35 | 0.35 | -0.03 | 0.08 |
| **TNF-α** | -0.65 | 0.05 | -0.10 | -0.37 | -0.33 | -0.38 | -0.33 |
| **IL-6** | -0.35 | 0.45 | 0.00 | -0.60 | 0.00 | 0.18 | 0.33 |
| **Pro-Infl. PC 1** | -0.50 | -0.14 | 0.03 | -0.47 | 0.03 | -0.58 | -0.38 |
| **IL-1RA** | -0.57 | 0.40 | -0.18 | -0.60 | -0.23 | 0.27 | 0.22 |
| **IL-10** | -0.24 | 0.39 | -0.79* | -0.71 | -0.71 | 0.76* | 0.64 |
| **Anti-Infl. PC 1** | -0.24 | 0.75 | -0.69 | -0.64 | -0.38 | 0.81* | 0.81* |

**D.**

| **AUD – Abstinent (N=10)** | **BDNF** | **GLP-1** | **Ghrelin** | **GIP** | **GH** | **Leptin** | **Insulin** |
| --- | --- | --- | --- | --- | --- | --- | --- |
| **IL-8** | -0.75* | 0.40 | -0.05 | -0.14 | 0.22 | -0.16 | 0.44 |
| **IL-18** | 0.10 | -0.12 | 0.38 | 0.00 | 0.21 | 0.33 | 0.07 |
| **CCL2** | -0.45 | 0.07 | -0.21 | 0.12 | -0.48 | -0.28 | 0.76* |
| **TNF-α** | -0.58 | 0.24 | -0.29 | 0.55 | 0.04 | 0.09 | 0.37 |
| **IL-6** | 0.12 | 0.76* | 0.05 | 0.45 | 0.37 | 0.16 | -0.09 |
| **Pro-Infl. PC 1** | -0.66* | 0.31 | -0.14 | 0.33 | -0.03 | 0.05 | 0.58 |
| **IL-1RA** | -0.03 | 0.36 | -0.05 | 0.71 | 0.18 | 0.25 | 0.13 |
| **IL-10** | -0.04 | -0.10 | 0.01 | 0.86* | -0.21 | -0.05 | -0.05 |
| **Anti-Infl. PC 1** | -0.20 | 0.19 | -0.21 | 0.83* | 0.03 | 0.03 | 0.08 |

Table S3 shows the Spearman’s r values for each protein x protein pair. Data from (A) combined groups (HC, CD, and AB groups combined), (B) Healthy Controls, (C) Currently Drinking AUD group, and (D) Abstinent AUD group. * p ≤ 0.05.

**SUPPLEMENTAL RESULTS**

**Figure S1 – Immune proteins over the course of the six study visits.**

Immune biomarker concentrations over the course of the six study visits. Biomarkers concentrations remained relatively stable over the course of the six study visits.

**Figure S2 – Endocrine proteins over the course of the six study visits.**

Endocrine biomarker concentrations over the course of the six study visits. Biomarkers concentrations remained relatively stable over the course of the six study visits.

**Figure S3 – Complete blood count (CBC)**

| Tests | Units | Reference Interval | HC | CD | AB | p-value |
| --- | --- | --- | --- | --- | --- | --- |
| WBC | x10E3/µL | 3.4 - 10.8 | 6.17 ± 1.84 | 6.40 ± 1.84 | 6.21 ± 3.01 | 0.97 |
| RBC | x10E6/µL | 3.77 - 5.28 | 4.9 ± 0.39 | 4.56 ± 0.46 | 4.67 ± 0.34 | 0.16 |
| Hemoglobin | g/dL | 11.2 - 15.9 | 14.58 ± 1.3 | 14.08 ± 1.13 | 14.08 ± 1.6 | 0.61 |
| Hematocrit | % | 34.0 - 46.6 | 43.74 ± 3.14 | 41.49 ± 3.33 | 42.36 ± 3.80 | 0.33 |
| MCV | fL | 79 - 97 | 89.49 ± 4.13 | 91.2 ± 4.35 | 91.03 ± 8.37 | 0.76 |
| MCH | pg | 26.6 - 33.0 | 29.81 ± 1.59 | 30.97 ± 1.91 | 30.26 ± 3.73 | 0.59 |
| MCHC | g/dL | 31.5 - 35.7 | 33.32 ± 1.06 | 33.92 ± 0.87 | 33.19 ± 1.47 | 0.36 |
| Platelets | x10E3/µL | 150 - 450 | 244.33 ± 43.44 | 248.00 ± 60.72 | 214.8 ± 72.81 | 0.4 |
| MPV | fL | 7.4 - 10.4 | 9.84 ± 0.49 | 9.97 ± 0.73 | 11.0 ± 1.21 | 0.0079* |
| Neutrophils (%) | % | not est. | 53.11 ± 13.26 | 58.03 ± 4.81 | 53.21 ± 10.77 | 0.52 |
| Lymphocytes (%) | % | not est. | 35.5 ± 13.3 | 27.82 ± 2.80 | 33.79 ± 9.97 | 0.23 |
| Monocytes (%) | % | not est. | 7.6 ± 2.4 | 9.68 ± 2.73 | 9.17 ± 2.76 | 0.12 |
| Eosinophils (%) | % | not est. | 2.6 ± 2.02 | 3.4 ± 2.19 | 2.79 ± 1.35 | 0.62 |
| Basophils (%) | % | not est. | 0.73 ± 0.45 | 0.71 ± 0.22 | 0.69 ± 0.26 | 0.96 |
| N-L Ratio (%) | n/a | not est. | 1.83 ± 1.06 | 2.12 ± 0.38 | 1.78 ± 0.82 | 0.64 |
| Neutrophils (Abs) | x10E3/µL | 1.4 - 7.0 | 3.33 ± 1.46 | 3.73 ± 1.19 | 3.53 ± 2.19 | 0.86 |
| Lymphocytes (Abs) | x10E3/µL | 0.7 - 3.1 | 2.17 ± 1.13 | 1.79 ± 0.58 | 1.93 ± 0.76 | 0.62 |
| Monocytes (Abs) | x10E3/µL | 0.1 - 0.9 | 0.45 ± 0.17 | 0.59 ± 0.14 | 0.53 ± 0.24 | 0.23 |
| Eosinophils (Abs) | x10E3/µL | 0.0 - 0.4 | 0.16 ± 0.16 | 0.21 ± 0.14 | 0.15 ± 0.07 | 0.58 |
| Basophils (Abs) | x10E3/µL | 0.0 - 0.2 | 0.042 ± 0.019 | 0.047 ± 0.023 | 0.043 ± 0.021 | 0.86 |

Complete blood count (CBC) data from blood collected on the first study visits. Values presented as group mean ± std. dev. for each group. The p-value is from a 1-Way ANOVA with group as the between subjects factor. WBC = White Blood Cell Count. RBC = Red Blood Cell Count. MCV = Mean Corpuscular Volume. MCH = Mean Corpuscular Hemoglobin. MCHC = Mean Corpuscular Hemoglobin Concentration. MPV = Mean Platelet Volume
